# Timely Initiation of Breastfeeding and Associated Factors in 20 Sub-Saharan African Countries: Cross-Sectional Multilevel Analysis of Recent Demographic and Health Surveys

**DOI:** 10.2196/103827

**Published:** 2026-10-19

**Authors:** Tadele Derbew Kassie, Getnet Gedif, Mequanent Dessie Bitewa, Atsede Alle Ewunetie, Tewodros Shibabaw, Menberu Gete, Melkamu Siferih, Mikias Getahun Molla, Tirusew Wondie Wodie, Daniel Mulat Eshetu, Abebaw Abeje Muluneh, Aysheshim Asnake Abneh

**Affiliations:** 1 Department of Health Promotion and Behavioral Science, College of Medicine and Health Science, Bahir Dar University, Bahir Dar, 273, Ethiopia, +251 936987005; 2 Institute of Public Health, College of Medicine and Health Sciences, University of Gondar, Gondar, Ethiopia; 3 Public Health Department, College of Medicine and Health Science, Debre Markos University, Amhara, Ethiopia; 4 School of Medicine, College of Medicine and Health Sciences, University of Gondar, Gondar, Ethiopia; 5 College of Medicine and Health Science, Debre Markos University, Amhara, Ethiopia; 6 Debre Markos University, Debre Markos, Amhara, Ethiopia; 7 Injibara University, Injibara, Amhara, Ethiopia; 8 Department of Midwifery, College of Medicine and Health Science, Debre Markos University, Debre Markos, Ethiopia

**Keywords:** timely initiation breastfeeding, associated factor, sub-Saharan Africa, demographic health survey, DHS

## Abstract

**Background:**

An important strategy to decrease infant morbidity and mortality is timely initiation of breastfeeding (TIBF), which is defined as putting the newborn to the breast within 1 hour of delivery. Delayed initiation of breastfeeding may contribute to high neonatal and infant mortality in sub-Saharan Africa.

**Objective:**

This study assessed the prevalence of TIBF and associated factors using recent Demographic and Health Survey data from 20 sub-Saharan African countries.

**Methods:**

Using recent Demographic and Health Survey data from sub-Saharan African countries, a cross-sectional study was conducted. Data were accessed on January 27, 2026. The analysis included 177,988 children after application of sampling weights. The hierarchical structure of the data was taken into consideration by conducting a multilevel logistic regression analysis. Results were presented using adjusted odds ratios (AORs) with 95% CIs. To evaluate cluster-level variance, random-effects measures such as the median odds ratio and intraclass correlation coefficient were reported.

**Results:**

Among 177,988 weighted samples, 59.05% (95% CI 58.82%-59.28%) initiated breastfeeding within 1 hour of birth. TIBF was more likely among rural residents (AOR 1.25, 95% CI 1.21‐1.28), mothers with primary (AOR 1.48, 95% CI 1.45-1.52), secondary (AOR 1.22, 95% CI 1.19-1.26), and higher education (AOR 1.22, 95% CI 1.15-1.28), institutional delivery (AOR 1.40, 95% CI 1.36-1.43), multiparous (AOR 1.19, 95% CI 1.15-1.23), and grand multiparous mothers (AOR 1.10, 95% CI 1.05-1.14). Lower odds were observed among cesarean births (AOR 0.31, 95% CI 0.30-0.33), twin births (AOR 0.87, 95% CI 0.82-0.92), delayed antenatal care initiation (AOR 0.84, 95% CI 0.82-0.85), female children (AOR 0.90, 95% CI 0.88-0.91), and mothers with a history of child death (AOR 0.90, 95% CI 0.86-0.94).

**Conclusions:**

TIBF remains suboptimal in sub-Saharan Africa and is influenced by maternal, obstetric, and health care factors. Enhancing antenatal counseling and postnatal breastfeeding support, particularly after cesarean delivery and among vulnerable groups, may improve timely initiation practices.

## Introduction

Timely initiation of breastfeeding (TIBF) is defined as placing the newborn to the breast within the first hour of delivery. This practice is strongly recommended by the World Health Organization (WHO), as it plays a vital role in promoting optimal infant and young child feeding. This behavior is favorable for the provision of colostrum to the infant, which contains high concentrations of immunoglobulin and other bioactive constituents, including growth factors that are essential for optimal neonatal nutrition, growth, development, and the establishment of passive immunity [1,2]. During the first 6 months of life, breast milk is the primary and most appropriate source of nutrition for newborns. It should be initiated within the first hour after birth, ideally even before the placenta is delivered, in line with recommendations from the WHO [2-4]. TIBF has the following advantages for infants: it decreases upper respiratory tract infections, promotes the development of dentition, enhances cognitive development, and reduces childhood obesity [5]. TIBF helps to reduce the risk of postpartum hemorrhage by promoting uterine contraction and faster uterine involution, supports quicker physical recovery after delivery, and strengthens mother-infant bonding [3,5,6]. It stimulates breast milk production and helps transfer protective antibodies to the infant, strengthening early immunity. For mothers, it reduces the risk of postpartum hemorrhage and supports quicker uterine recovery. It also promotes emotional attachment and strengthens the mother-child bond [7].

The Baby-Friendly Hospital Initiative (BFHI), established by the WHO and United Nations Children’s Fund (UNICEF) in 1991, developed a platform of 10 steps to improve maternal and neonatal care in health care institutions and promote optimal breastfeeding practices, including TIBF [8].

Worldwide, the magnitude of TIBF ranged from 17.7% to 98.4%, with an average magnitude of 57.6%. A global study demonstrated that an estimated 78 million newborns were not put to the breast within the first hour of delivery, based on a joint report by the UNICEF and the WHO [3,8]. A pooled analysis of Economic Community of West African States countries found that the magnitude of TIBF was 43%, highlighting notable differences in breastfeeding practices across the region [9].

Delayed initiation of breastfeeding (DIBF) is highly practiced, and reports have highlighted that it is linked with increased neonatal morbidity and mortality, particularly in low- and middle-income countries [10]. The 2019 mini-demography health survey of Ethiopia revealed that the prevalence of TIBF was 73.6% [11]. Evidence from sub-Saharan African countries from the 2013 Demographic and Health Survey (DHS) revealed that the prevalence of TIBF was 57% [12] and 34.7% [13], which were below the recommendations.

Maternal complications during pregnancy and cesarean delivery were associated with DIBF. Health facility deliveries that adhered to postnatal care protocols promoted TIBF among mothers [8,9,12]. Antenatal care attendance, having female infants, urban residence, multiparity, larger birth size, maternal unemployment status, and belonging to wealthier households were significantly associated with TIBF [9,11,12].

Although there are different recommendations by the WHO and UNICEF about the importance of TIBF within 1 hour, many newborns are not put to the breast within the recommended time [14]. Nowadays, different evidence demonstrates that TIBF reduces infant mortality by 22% [15]. Countries in sub-Saharan Africa had an infant mortality rate of 52 per 100 live births, which is higher than the global level, and this could be reduced by TIBF [12].

While the national policies and recommendations in Africa advocate and promote timely breastfeeding initiation, countries are not adhering to these recommendations [6,7,13,16]. There are other programs that promote TIBF in African countries, such as the national policy on infant and young child feeding, the WHO, and the Community-Integrated Management of Childhood Illness [2,17].

Reports from previous studies on the continent demonstrated that TIBF is complex and shaped by many factors, notably maternal sociodemographic characteristics like age, education, and residence, as well as use of maternal health services, including antenatal care visits and place of delivery [18].

Countries in sub-Saharan Africa continue to face high neonatal and infant mortality rates, and one contributing factor could be the DIBF after birth. There is a need to determine the updated pooled estimate level of TIBF and its key determinants at the continent level. Recent DHS data from 20 sub-Saharan countries can provide important information to estimate the magnitude and identify factors. Therefore, the aim of this study was to assess the prevalence of TIBF and to identify factors associated with this among women who delivered in the last 5 years preceding the survey in the selected countries, using data from the recent DHS.

## Methods

### Study Design and Setting

The study included 20 countries in sub-Saharan Africa that had recent Demographic and Health Survey data available. The countries were selected based on the availability of recent DHS datasets and included Angola, Burkina Faso, the Democratic Republic of the Congo, Côte d'Ivoire, Gabon, Ghana, The Gambia, Kenya, Liberia, Lesotho, Madagascar, Mali, Mauritania, Mozambique, Nigeria, Rwanda, Sierra Leone, Senegal, Tanzania, and Zambia.

### Data Source and Extraction

We obtained the required authorization to access the dataset online after submitting a request for the data via the DHS online platform, accompanied by a detailed explanation of the study’s objectives and research methodology. The data were accessed using the following login link [19].

### Populations

The study population comprised children aged 6 to 59 months from 20 sub-Saharan African countries with available DHS data conducted between 2019 and 2024. We used the DHS Kids’ Recode files for the analysis. The countries included in the study, their respective regions, DHS survey periods, and sample sizes are presented in Table 1. Countries were eligible for inclusion if they had a DHS conducted from 2019 onward and the available dataset contained the variables required for the present analysis. Countries without an eligible DHS conducted during the study period, including those whose most recent DHS was conducted before 2019, were excluded.

**Table 1.** Countries, regions, Demographic and Health Survey periods, and sample sizes included in the analysis.

Region and country	DHS^a^ dataset	Survey period	Sample size, n (%)
West Africa			
Burkina Faso	BF8	2021	8277 (4.65)
Côte d’Ivoire	CI8	2021	6599 (3.71)
Ghana	GH8	2022‐2023	6429 (3.61)
The Gambia	GM7	2019‐2020	7494 (4.21)
Liberia	LB7	2019‐2020	5087 (2.86)
Mali	ML8	2023‐2024	12,667 (7.12)
Mauritania	MR7	2019‐2021	11,188 (6.29)
Nigeria	NG8	2023‐2024	22,542 (12.66)
Senegal	SN8	2023	6830 (3.84)
Sierra Leone	SL7	2019	9461 (5.32)
Central Africa			
Democratic Republic of the Congo	CD8	2023‐2024	17,173 (9.65)
Gabon	GA7	2019‐2021	5639 (3.17)
East Africa			
Kenya	KE8	2022	6594 (3.7)
Madagascar	MD7	2021	11,989 (6.74)
Rwanda	RW7	2019‐2020	8205 (4.61)
Tanzania	TZ8	2022	8365 (4.7)
Southern Africa			
Angola	AO8	2023‐2024	8092 (4.55)
Lesotho	LS8	2023‐2024	1548 (0.87)
Mozambique	MZ8	2022‐2023	7647 (4.3)
Zambia	ZM8	2024	6162 (3.46)
Total (20 countries)	—^b^	2019‐2024	177,988 (100)

a DHS: Demographic and Health Surveys.

b Not applicable.

### Sample Size Determination and Sampling Procedure

#### Sample Size Determination

The sample size for this study included all children who were born within the last 5 years preceding the survey and were documented in the dataset. After eliminating and adjusting for relevant variables and applying sampling weights, a total weighted sample of 177,988 was extracted and included in the analysis.

#### Sampling Procedure

The DHS used a stratified 2-stage sample design. Stratification was achieved by separating each of the countries’ states and the Federal Capital Territory into urban and rural areas. Samples were selected independently in every stratum via a 2-stage selection. The first stage involved selecting sample points (clusters) consisting of enumeration areas. Enumeration areas were drawn with probability proportional to their size within each sampling stratum.

### Variables of the Study

#### Dependent Variable

The primary outcome variable of the study is the magnitude of TIBF in 20 sub-Saharan African countries.

#### Independent Variables

These were categorized as individual-level factors and community-level factors, as described below:

- Individual-level factors: maternal age, educational level of the mother, media exposure (mass media and print), parity, wanted the last child, sex of household head, ever had died children, wanted pregnancy, getting permission to visit a medical health facility, transport cost, respondent’s occupation.
- Health sector–related factors: mode of delivery (delivered by cesarean section or vaginal birth), ever had history of terminated pregnancy (abortion)
- Community-level factors: residence, distance from health facility, and wealth index

### Operational Definition

#### TIBF

It was defined as the proportion of children aged 0 to 59 months who were put to the breast within the first hour of delivery [20].

#### Parity

This is the total number of live births that a woman has given birth to. It was classified as primiparous (1 birth), multiparous (2‐5 births), and grand multiparous (>5 births), consistent with prior DHS analyses [21].

#### Media Exposure

If a woman reads, listens to, or watches mass media (radio, TV, and newspaper) at least once a week, she is considered to have media exposure [22].

### Data Management, Processing, and Analysis Methods

Secondary data obtained from the DHS survey were used, and the extracted dataset was analyzed. The data were obtained from an online source. They were cleaned, recoded, and analyzed using Stata (version 17; StataCorp). In line with DHS survey recommendations, sampling weights were applied to adjust for sampling probabilities and nonresponse, ensuring nationally representative and statistically reliable estimates before analysis.

Bivariable logistic regression followed by multivariable logistic regression analyses was conducted. Variables with a *P* value less than .25 in the bivariable regression were considered for inclusion in the multivariable logistic regression model. Both crude odds ratios and adjusted odds ratios (AORs) with 95% CIs were calculated. Variables with *P* values less than .05 were considered statistically significant. Three models were constructed: Model I included individual-level variables, Model II included community-level variables, and Model III (final model) included both individual- and community-level variables simultaneously. Multicollinearity among independent variables was assessed using the variance inflation factor (VIF). A VIF value greater than 10 was considered evidence of multicollinearity. To obtain statistically valid regression coefficient estimates, clustering information was used.

A multilevel analysis was employed to account for both person-level and community-level predictors, with a random effect for variations between clusters. Model I included individual-level factors, Model II included community-level factors, and Model III included both individual- and community-level factors. The significance of the random intercept variation was assessed using the likelihood ratio test. Measures of variation for the random effect between clusters were determined using the intraclass correlation coefficient (ICC), proportional change in variance, and median odds ratio (MOR).

### Ethical Considerations

Ethical approval for data collection was obtained from the relevant institutional review boards overseeing the DHS Program in each participating country. Permission to use the dataset was requested from the DHS Program, and approval was obtained. The institutional review board–approved procedures for DHS public-use datasets do not in any way allow respondents, households, or sample communities to be identified. There are no names of individuals or household addresses in the data files. This study analyzed publicly available, deidentified secondary data and was therefore exempt from further ethical review. All analyses were conducted in accordance with established ethical standards of the Declaration of Helsinki.

## Results

### Sociodemographic and Other Factors

A total of 177,988 weighted samples were extracted and included from the DHS Kids’ Record dataset. The mean age of the respondents was 28.90 (95% CI 28.86‐28.93) years. Among the study participants, 64,260 (36.1%) were from urban residences. The majority of households were male-headed (n=140,983, 79.21%), and regarding age distribution, 70.59% (n=125,642) of women were in the age group of 20 to 34 years. Concerning media exposure, more than half (n=90,233, 50.7%) had media exposure (Table 1).

The largest contribution to the pooled sample came from Nigeria (NG8), accounting for 12.66% (22,542/177,988) of the total population, with 14,106 late initiations and 8436 timely initiations. This was followed by the Democratic Republic of the Congo (CD8), which contributed 9.65% (17,173/177,988) of the sample, including 7387 late and 9786 timely initiations. In contrast, Lesotho (LS8) contributed the smallest proportion (1548/177,988, 0.87%) with 442 late initiations and 1106 timely initiations (Table 2).

**Table 2.** Sociodemographic and other factors for the timely initiation of breastfeeding evidenced from the Demographic and Health Surveys of 20 sub-Saharan African countries.

Variables and categories	Late	Timely	Total (%)
Place of residence			
Urban	28,208	36,052	36.1
Rural	44,671	69,056	63.9
Sex of head of the household			
Male	58,679	82,304	79.21
Female	14,201	22,804	20.79
Women’s educational level			
No education	27,783	35,364	35.48
Primary	18,467	33,524	29.21
Secondary	22,625	31,518	30.42
Higher and above	4005	4703	4.89
Media exposure			
No media exposure	35,196	52,560	49.3
Has media exposure	37,684	52,549	50.7
Respondents occupation			
Has no job	27,902	39,955	38.12
Has job	44,977	65,153	61.88
Wealth index			
Poor	32,845	49,475	46.25
Middle	14,390	21,180	19.98
Rich	25,644	34,453	33.76
Respondents’ age in years			
15‐19	4929	6638	6.5
20‐34	51,910	73,732	70.59
35‐49	16,041	24,738	22.91
Place of delivery			
Home delivery	16,526	20,108	20.58
Institutional delivery	56,354	84,999	79.42
Delivered by cesarean section			
No	64,310	100,368	92.52
Yes	8569	4741	7.48
Sex of the child			
Male	42,139	63,611	59.41
Female	30,740	41,498	40.59
Had history of abortion			
No	59,134	85,394	81.2
Yes	13,745	19,714	18.8
Getting permission to go to medical help			
Not a big problem	47,782	67,201	64.6
Big problem	25,097	37,907	35.4
Distance to medical health facility			
Not a big problem	28,306	44,562	40.94
Big problem	44,573	60,546	59.06
Parity			
Primiparous	11,972	15,481	15.42
Multiparous	37,927	56,817	53.23
Grand multiparous	22,980	32,810	31.35
The child is a twin			
No (single)	70,089	101,989	96.68
Yes (twin)	2789	3120	3.32
Had ever died children			
No	67,934	98,807	93.68
Yes	4945	6302	6.32

### The Magnitude of TIBF Among Sub-Saharan African Countries

The magnitude of TIBF was 59.05% (95% CI 58.82%‐59.28%) within 1 hour of delivery.

### Factors Associated With the TIBF

All included individual- and community-level variables were candidates for multivariable logistic regression as they had a *P* value less than .25. Individual-level and community-level factors were entered into separate regression models initially, followed by a final model (Model III) in which both sets of factors were included simultaneously. The analysis identified several factors associated with the TIBF. In the final model (Model III), type of place of residence, sex of head of household, sex of the child, women’s educational level, media exposure, respondents’ occupation, respondents’ age, place of delivery, getting permission to seek medical help, distance to a health facility, initiation of first antenatal care, parity, twin status of the child, delivery by cesarean section, and history of child death were significantly associated with TIBF (*P*<.05).

Mothers living in rural areas had higher odds of TIBF compared with urban residents (AOR 1.25, 95% CI 1.21‐1.28). Maternal education showed a positive association, where women with primary education had 48% (AOR 1.48, 95% CI 1.45‐1.52) compared to no education, secondary education has 22% (AOR 1.22, 95% CI 1.19‐1.26), and higher education and above had 22% (AOR 1.22, 95% CI 1.15‐1.28) more likely to initiate breastfeeding timely compared with women with no education. Mothers who delivered by cesarean section had reduced odds of TIBF (AOR 0.31, 95% CI 0.30‐0.33). Similarly, mothers of twin births had significantly lower odds of TIBF compared with singleton births (AOR 0.87, 95% CI 0.82‐0.92). Multiparous and grand multiparous mothers were more likely to timely initiate breastfeeding (AOR 1.19, 95% CI 1.15‐1.23 and AOR 1.10, 95% CI 1.05‐1.14, respectively). Mothers who initiated their first ANC visit late had lower odds of TIBF (AOR 0.84, 95% CI 0.82‐0.85), while mothers who delivered at a health institution had higher odds of TIBF (AOR 1.40, 95% CI 1.36‐1.43) (Table 3).

**Table 3.** Distribution of timing of breastfeeding initiation across selected sub-Saharan African countries.

Country	Late (n)	Timely (n)	Total (%)
Angola (AO8)	3477	4615	4.55
Burkina Faso (BF8)	2869	5408	4.65
Democratic Republic of the Congo (CD8)	7387	9786	9.65
Côte d'Ivoire (CI8)	3487	3112	3.71
Gabon (GA7)	1378	4261	3.17
Ghana (GH8)	2566	3863	3.61
The Gambia (GM7)	4694	2800	4.21
Kenya (KE8)	2593	4001	3.70
Liberia (LB7)	1694	3393	2.86
Lesotho (LS8)	442	1106	0.87
Madagascar (MD7)	4409	7580	6.74
Mali (ML8)	6627	6040	7.12
Mauritania (MR7)	4664	6524	6.29
Mozambique (MZ8)	1465	6182	4.30
Nigeria (NG8)	14,106	8436	12.66
Rwanda (RW7)	1093	7112	4.61
Sierra Leone (SL7)	2100	7361	5.32
Senegal (SN8)	3937	2893	3.84
Tanzania (TZ8)	2033	6332	4.70
Zambia (ZM8)	1857	4305	3.46
Total	72,879	105,108	100

### Random Effects and Model Comparisons

In the empty (null) model, the ICC revealed that variations between cluster areas accounted for 8.3% (ICC 0.083) of the overall variation in TIBF after delivery, while individual differences accounted for the remaining 91.7% of the variability (Table 4). In the null model, the odds of TIBF differed between higher- and lower-risk clusters by a factor of 1.68 (MOR 1.68).

**Table 4.** Factors associated with timely initiation of breastfeeding among children in sub-Saharan Africa, evidence from the Demographic and Health Surveys.

Variables and categories	Model I, AOR^a^ (95% CI)	Model II, AOR (95% CI)	Model III, AOR (95% CI)
Type of place of residence			
Urban	—^b^	1	1
Rural	—	1.21 (1.18‐1.24)	1.25 (1.21‐1.28)^c^
Sex of head of the household			
Male	1	—	1
Female	1.13 (1.10‐1.16)^c^	—	1.14 (1.12‐1.17)^c^
Sex of the child			
Male	1	—	1
Female	0.90 (0.88‐0.92)^c^	—	0.90 (0.88‐0.91)^c^
Women’s educational level			
No education	1	—	1
Primary	1.48 (1.44‐1.52)^c^	—	1.48 (1.45‐1.52)^c^
Secondary	1.17 (1.13‐1.20)^c^	—	1.22 (1.19‐1.26)^c^
Higher and above	1.12 (1.07‐1.18)^c^	---	1.22 (1.15‐1.28)^c^
Media exposure			
No media exposure	1	—	1
Has media exposure	0.93 (0.91‐0.95)^c^	—	0.97 (0.95‐0.99)^c^
Respondents’ occupation			
Has no job	1	—	1
Has job	0.98 (0.96‐1.01)	—	0.97 (0.95‐0.99)^c^
Wealth index			
Poor	—	1	1
Middle	—	1.04 (1.02‐1.07)	1.00 (0.97‐1.03)
Rich	—	1.04 (1.01‐1.07)	1.02 (0.99‐1.05)
Respondents’ age in years			
15‐19	1	—	1
20‐34	1.06 (1.02‐1.11)^c^	—	1.07 (1.03‐1.12)^c^
35‐49	1.24 (1.18‐1.31)^c^	—	1.26 (1.20‐1.33)^c^
Place of delivery			
Home delivery	1	—	1
Institutional delivery	1.37 (1.33‐1.40)^c^	—	1.40 (1.36‐1.43)^c^
Getting permission to go to medical help			
Not a big problem	1	—	1
Big problem	0.98 (0.96‐1.01)	—	0.96 (0.93‐0.99)^c^
Distance to medical health facility			
Not a big problem	—	1	1
Big problem	—	1.03 (1.00‐1.05)	1.04 (1.02‐1.07)^c^
Initiation of first antenatal care			
Early	1	—	1
Late	0.84 (0.82‐0.86)^c^	—	0.84 (0.82‐0.85)^c^
Parity			
Primiparous	1	—	1
Multiparous	1.19 (1.15‐1.22)^c^	—	1.19 (1.15‐1.23)^c^
Grand multiparous	1.10 (1.06‐1.14)^c^	—	1.10 (1.05‐1.14)^c^
The child is twin			
Single	1	—	1
Twin	0.87 (0.82‐0.92)^c^	—	0.87 (0.82‐0.92)^c^
Delivered by cesarean section			
No	1	—	1
Yes	0.31 (0.30‐0.33)^c^	—	0.31 (0.30‐0.33)^c^
Had ever died children			
No	1	—	1
Yes	0.90 (0.86‐0.94)^c^	—	0.90 (0.86‐0.94)^c^

a AOR: adjusted odds ratio.

b Not applicable.

c *P*<.05.

Model fitness was assessed using the Akaike information criterion, Bayesian information criterion, and deviance statistics. The model with the lowest deviance value was considered the best-fitting model. Accordingly, the final model (Model III) had the lowest deviance (230,786.96) compared with the null model (236,767.84), Model I (231,114.1), and Model II (236,494.74), indicating better model fitness for identifying factors significantly associated with TIBF among children born within the 5 years preceding the DHS survey. Similarly, Model III had the lowest Akaike information criterion (230,833) and a relatively lower Bayesian information criterion (231,065.8) compared with the other fitted models.

In the final model (Model III), the cluster-level variation in TIBF remained at 8.3% (ICC 0.083), suggesting the presence of unobserved community-level factors influencing breastfeeding initiation practices. The MOR slightly decreased from 1.69 in Model I to 1.68 in Model III, indicating persistent but relatively stable cluster-level heterogeneity after controlling for both individual- and community-level factors. The proportional change in variance indicated that the variables included in the final model explained only a minimal proportion of the variation (−0.01%) in TIBF compared with the null model. In addition, the mean VIF (1.74) in Model III suggested no evidence of problematic multicollinearity among explanatory variables (Table 5).

**Table 5.** Model comparison for timely initiation of breastfeeding among children in sub-Saharan Africa: evidence from the Demographic and Health Surveys data^a^.

Parameters	Null model	Model I	Model II	Model III
AIC^b^	236,771.8	231,152.1	236,506.7	230,833
BIC^c^	236,792.1	231,344.4	236,567.5	231,065.8
Cluster level variance	0.2962864	0.3054656	0.2838114	0.2963245
ICC^d^	0.0826195	0.0849617	0.0794171	0.0826293
MOR^e^	1.680719	1.6941875	1.662252	1.6807751
PCV^f^	Reference	−3.10%	4.21%	−0.01%
Deviance	236,767.84	231,114.1	236,494.74	230,786.96
Mean VIF^g^	—	1.76	1.38	1.74

a *P*<.05.

b AIC: Akaike information criterion.

c BIC: Bayesian information criterion.

d ICC: intraclass correlation coefficient.

e MOR: median odds ratio.

f PCV: proportional change in variance.

g VIF: variance inflation factor.

## Discussion

### Pirncipal Findings

The analysis in these 20 sub-Saharan African countries showed that 59.05% (95% CI 58.82%‐59.28%) of children initiated breastfeeding within the first hour of delivery, indicating suboptimal TIBF. This finding was slightly higher than the pooled estimate of 58.3% reported from 35 sub-Saharan African countries between 2008 and 2019 [23]. This similarity could reflect persistent regional barriers, including inadequate breastfeeding counseling [24], limited access to maternal health care services [25], and sociocultural practices that delay timely breastfeeding initiation [26]. This increment might also be attributed to gradual improvements in maternal and child health services and breastfeeding promotion programs across the region, including BFHI programs, increased antenatal counseling, and expanded awareness campaigns emphasizing the importance of initiating breastfeeding within 1 hour of birth. These initiatives are known to improve mothers’ knowledge and attitudes toward timely breastfeeding [8,16,17].

The finding from the analysis in the included countries revealed that the magnitude of TIBF is lower than that reported in studies conducted in many sub-Saharan African countries. Higher magnitudes were reported in Ethiopia (73.6% from Mini DHS 2019), Burundi (85%), Uganda (71.6%), Malawi (76.9%), and Mozambique (75.03%) [11,13,27-29]. This variation may be attributed to the inclusion of pooled data from 20 sub-Saharan African countries with differences in health care systems, socioeconomic contexts, and breastfeeding promotion strategies, which could influence the overall estimate. Conversely, findings from individual countries may reflect more favorable maternal health services and country-specific progress in breastfeeding practices [13,30]. Higher rates of TIBF in some countries could be attributed to better implementation of maternal health care services, particularly skilled birth attendance and institutional delivery care [8]. Effective integration of breastfeeding promotion into antenatal care and immediate postpartum support, particularly early skin-to-skin contact and adherence to BFHI guidelines, contributed to significant improvements in the initiation of breastfeeding within 1 hour of delivery [10,31]. In settings where health facilities provide consistent postpartum breastfeeding support within the first hour of birth, higher rates of TIBF have been reported, highlighting the importance of strengthening immediate postnatal care practices across health facilities [32].

Community-based health promotion activities may explain the differences. Countries with strong community health worker programs that deliver breastfeeding counseling after delivery tend to achieve a higher magnitude of TIBF [33,34]. In contrast, poor counseling quality, shortages of trained health professionals in breastfeeding counseling, and absence of postnatal care can delay its initiation.

In contrast, the magnitude in the current study is higher than that in Guinea (16.6%), Congo-Brazzaville (24%), and Chad (23%) [13]. Such variations have also been documented in previous sub-Saharan African analyses, which show substantial between-country differences in TIBF, largely due to disparities in maternal health care access, socioeconomic status, and cultural practices influencing newborn feeding behaviors. Differences in study design, sample size, and sociodemographic composition may also contribute to these inconsistencies [17,30].

In this study, women from rural residences had higher odds of TIBF (AOR 1.25) compared with their counterparts. The higher odds of timely breastfeeding initiation among rural women may be attributed to urban health facilities, which may also have greater formula exposure and weaker adherence to Baby-Friendly practices. In addition, urban mothers are more likely to be influenced by modern feeding beliefs, face employment-related constraints, and experience inconsistent breastfeeding counseling despite better access to health services [17].

The present study findings reported that women with higher educational levels were more likely to initiate breastfeeding in a timely manner. This is supported by evidence from Ethiopia Demographic and Health Survey analyses, which show that mothers with secondary or higher education have significantly increased odds of timely breastfeeding initiation compared with uneducated women [25].

Women with media exposure demonstrated significantly decreased odds of TIBF in the current study. This finding is inconsistent with the study conducted in pooled sub-Saharan African countries using DHS data, which identified media exposure as a strong enabler of early initiation of breastfeeding [9,13]. This inconsistency may be due to differences in the type of media content accessed in the study setting, where exposure may include formula marketing or mixed infant feeding messages that discourage immediate breastfeeding. In contrast, in settings covered by DHS analyses, mass media often delivers coordinated public health campaigns promoting TIBF. Variations in media regulation and the effectiveness of breastfeeding promotion messages may therefore explain the differing associations [35].

Age of the mother was also a significant factor in TIBF. Women aged 20 to 34 years and 35 to 49 years had higher odds (AOR 1.07 and 1.26, respectively) compared with those aged 15 to 19 years, indicating an age-related disparity in TIBF. The possible reason for this is that young women could have relatively poor knowledge of the importance of timely breastfeeding and may not have received antenatal care or institutional delivery, which have a direct or indirect effect on TIBF [36]. This finding is similar to that of a study conducted in Nigeria [37]. Additionally, older women, particularly multiparous mothers, may rely on previous childbirth experience and perceived knowledge, which may positively influence TIBF. However, this pattern contrasts with studies reporting that younger mothers demonstrate higher timely breastfeeding behavior, possibly due to targeted maternal health programs, perceived vulnerability, and stronger exposure to health promotion messages [38].

Women who reported difficulty obtaining permission to seek medical care had lower odds of TIBF. This may be because restricted autonomy limits access to antenatal care, institutional delivery, and postnatal breastfeeding counseling, all of which positively influence timely breastfeeding initiation.

Women who delivered in health facilities were more likely to initiate breastfeeding timely than those who delivered at home. This finding is consistent with a study conducted in Ethiopia [39], which reported that institutionally delivered women had about 2 times higher odds of TIBF. This may be because health facility delivery provides access to skilled birth attendants, immediate breastfeeding counseling, and postpartum support, including early skin-to-skin contact, which facilitates breastfeeding within the first hour after birth [40].

The findings of this study indicated that multiparous and grand multiparous women were more likely to initiate breastfeeding within 1 hour of delivery compared with nulliparous mothers. This may be because first-time mothers often have limited experience and knowledge regarding breastfeeding, may feel uncertain about proper positioning of the baby, lack confidence to initiate breastfeeding immediately after birth, and require more guidance on breastfeeding techniques. They also experience stress and anxiety during labor and can delay skin-to-skin contact and breastfeeding initiation within the recommended hour. Nulliparous women may rely heavily on family advice, which is not always supportive of immediate breastfeeding. Elders may prioritize other newborn practices before breastfeeding, and misconceptions about colostrum may delay initiation of breastfeeding. This evidence is similar to that from studies conducted in Nepal, Bangladesh [41], Ghana, and Ethiopia [13]. This similar report could be because of the similarity in cultural perceptions and sociodemographic factors.

In this study, twin children had DIBF compared with singletons. This is because twin pregnancies are more likely to have preterm birth, low birth weight, and high fetal complications, more likely to have an elective cesarean section. These mentioned unfavorable outcomes lead for the DIBF [42,43].

Employed women had lower odds of TIBF compared with unemployed women. This may be related to work-related stress, fatigue, and competing responsibilities, which may reduce preparedness for immediate breastfeeding. Employed mothers may also be more exposed to formula-feeding alternatives and concerns about balancing breastfeeding with future work demands.

Women with a history of child death had lower odds of TIBF. This may reflect previous adverse birth experiences, maternal psychological distress, or underlying health and socioeconomic vulnerabilities that negatively influence maternal care practices, including timely breastfeeding initiation.

### Limitations

Some subnational areas had relatively small sample sizes, requiring cautious interpretation of findings. As a secondary analysis of DHS data, important factors such as cultural beliefs, psychosocial influences, partner preferences, and qualitative maternal perspectives were unavailable. In addition, breastfeeding initiation was self-reported for births occurring within the 5 years preceding the survey, which may introduce recall bias. Finally, the cross-sectional nature of the data limits causal inference between explanatory variables and TIBF.

### Conclusion and Recommendations

The TIBF according to the DHS survey in sub-Saharan African countries remains suboptimal. Although this reflects a modest improvement compared with previous surveys, it demonstrates inconsistent national progress toward global breastfeeding targets recommended by the WHO and United Nations Children’s Fund. Individual-level and community-level factors were regressed together in the final model, and place of residence, women’s educational level, media exposure, wealth index, age of the respondent, mode of delivery, wanted child, getting permission to go to a health facility, parity, and type of pregnancy were significantly associated with TIBF.

The substantial cluster-level variation suggests the influence of contextual and health system factors. Strengthening implementation of the BFHI, improving access to skilled delivery and postnatal counseling, particularly in rural areas, and promoting maternal education and social protection mechanisms are critical to enhancing timely breastfeeding practices in the country.

Rural-urban disparities highlight the need for expanded access to skilled birth attendance, quality antenatal counseling, and timely postnatal support in rural settings.

Educational and socioeconomic disparities observed in this study suggest that policies promoting female education, male partner involvement, media-based health promotion, and financial risk protection (including health insurance coverage) may enhance timely initiation practices. Strengthening family planning services may further contribute by reducing unintended pregnancies, which were associated with DIBF.

## Abbreviations

AOR: adjusted odds ratio
BFHI: Baby-Friendly Hospital Initiative
DHS: Demographic and Health Survey
DIBF: delayed initiation of breastfeeding
ICC: intraclass correlation coefficient
MOR: median odds ratio
TIBF: timely initiation of breastfeeding
UNICEF: United Nations Children’s Fund
VIF: variance inflation factor
WHO: World Health Organization
